# A novel method for the *in vivo* isolation of circulating tumor cells from peripheral blood of cancer patients using a functionalized and structured medical wire

**DOI:** 10.3892/ijo.2012.1557

**Published:** 2012-07-16

**Authors:** NADIA SAUCEDO-ZENI, STEFFI MEWES, ROBERT NIESTROJ, LUKASZ GASIOROWSKI, DAVID MURAWA, PIOTR NOWACZYK, TATIANA TOMASI, EKKEHARD WEBER, GRZEGORZ DWORACKI, NILS G. MORGENTHALER, HEIKE JANSEN, CORINNA PROPPING, KAROLINA STERZYNSKA, WOJCIECH DYSZKIEWICZ, MACIEJ ZABEL, MARION KIECHLE, UTE REUNING, MANFRED SCHMITT, KLAUS LÜCKE

**Affiliations:** 1 GILUPI GmbH, Potsdam, Germany;; 2 Department of Thoracic Surgery, WCPiT, Poznan University of Medical Science;; 3 First Department of Surgical Oncology and General Surgery, Wielkopolska Cancer Center, 61-866 Poznan, Poland;; 4 Department of Obstetrics and Gynecology, Klinikum rechts der Isar, Technische Universitaet Muenchen, Munich, Germany;; 5 Poznan University of Medical Science, Fredry 10, 61-701 Poznan, Poland

**Keywords:** circulating tumor cells, breast cancer, non-small cell lung cancer, epithelial cell adhesion molecule, medical wire

## Abstract

The isolation of circulating tumor cells (CTCs) from the blood of patients afflicted with solid malignant tumors becomes increasingly important as it may serve as a ‘liquid biopsy’ with the potential of monitoring the course of the cancer disease and its response to cancer therapy, with subsequent molecular characterization. For this purpose, we functionalized a structured medical Seldinger guidewire (FSMW), normally used to obtain safe access to blood vessels and other organ cavities, with a chimeric monoclonal antibody directed to the cell surface expressed epithelial cell surface adhesion molecule (EpCAM). This medical device was optimized *in vitro* and its biocompatibility was tested according to the regulations for medical devices and found to be safe with no noteworthy side effects. Suitability, specificity and sensitivity of the FSMW to catch and enrich CTCs *in vivo* from circulating peripheral blood were tested in 24 breast cancer or non-small cell lung cancer (NSCLC) patients and in 29 healthy volunteers. For this, the FSMW was inserted through a standard venous cannula into the cubital veins of healthy volunteers or cancer patients for the duration of 30 min. After removal, CTCs were identified by immunocytochemical staining of EpCAM and/or cytokeratins and staining of their nuclei and counted. The FSMW successfully enriched EpCAM-positive CTCs from 22 of the 24 patients, with a median of 5.5 (0–50) CTCs in breast cancer (n=12) and 16 (2–515) CTCs in NSCLC (n=12). CTCs could be isolated across all tumor stages, including early stage cancer, in which distant metastases were not yet diagnosed, while no CTCs could be detected in healthy volunteers. In this observatory study, no adverse effects were noted. Evidently, the FSMW has the potential to become an important device to enrich CTCs *in vivo* for monitoring the course of the cancer disease and the efficacy of anticancer treatment.

## Introduction

Personalized cancer treatment is at present one of the most challenging goals in cancer research in order to improve health and quality of life of cancer patients (1,2). Since many tumor cells are distinct on the molecular level (3), and modern cancer drugs target selected molecular pathways, the definitive goal is to identify cancer patients at risk and those who may benefit from a certain cancer therapy (1,2). For this purpose, the isolation of circulating tumor cells (CTC) from the peripheral blood of patients afflicted with cancer becomes increasingly important and thus is in the focus of cancer research and the pharmaceutical industry (4). Enrichment and enumeration of CTC offer the potential as a prognostic cancer biomarker and may fulfill the criteria for a surrogate biomarker to evaluate the response of patients to cancer therapy (5,6). Molecular characterization of CTC is a rapidly developing research field aiming at revealing novel drug targets and to investigate mechanisms of tumor metastasis (7).

Over the past decade, several *in vitro* methodological approaches to isolate and detect rare CTC in the peripheral blood of cancer patients have been reported, including flow cytofluorometry (8), image-based immunological approaches (9), fluidic microchip technology (10), and PCR methods (11–13). At present, an antibody-coated magnetic particle isolation system targeting the epithelial cell surface EpCAM is used in most studies designed for *ex vivo* quantification of CTC in the blood of patients with advanced breast, colon, or prostate cancer (14–16). Patients with metastasized cancer diseases exhibit detectable numbers of CTC in their blood (17), however, since all *ex vivo* detection systems are limited by the blood volume that can be obtained from the patients or handled by the detection system (18), these technologies are of relatively low sensitivity.

To overcome the limitations of small blood sample volumes of the *ex vivo* CTC isolation techniques, new approaches are needed for screening large blood volumes *in vivo* using for example the principles of photoacoustic flow cytofluorometry (19). We developed an alternative medical device: a structured and functionalized medical wire (FSMW) based on a Seldinger guidewire (20) that offers the opportunity of capturing CTC from the circulating blood of cancer patients. Like in other *ex vivo* CTC-detection technologies, identification of CTC captured by the FSMW is performed by phenotyping CTC with antibodies directed to cytokeratins and/or epithelial cell markers. Occasionally trapped hematologic cells are identified by antibodies directed to respective hematologic cell surface markers. Here, we describe a novel *in vivo* CTC-catching medical device, the FSMW, its biocompatibility and first results of its application for *in vivo* enrichment of CTC from the peripheral blood of patients presenting with breast cancer or non-small cell lung cancer (NSCLC).

## Materials and methods

### Functionalized structured medical wire

**Figure 1:**
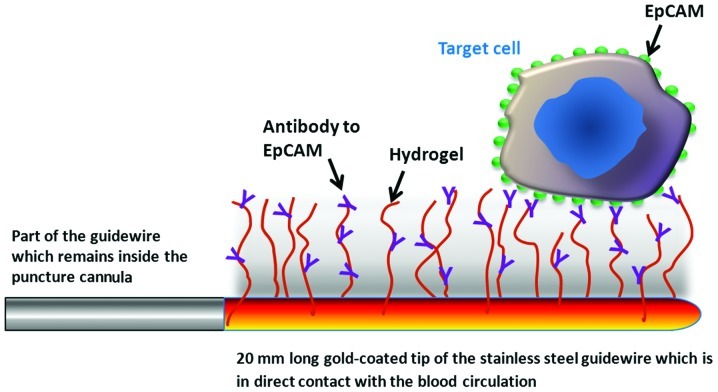
Schematic drawing of the functionalized tip of the FSMW. Antibodies to the epithelial cell surface antigen EpCAM are attached to a polycarboxylate hydrogel (1–5 μm) which is coated on a gold-plated (200 nm) Seldinger guidewire. Then the hydrogel is functionalized with antibodies to the EpCAM. This FSMW interacts with target cells expressing EpCAM antigen on their surface, e.g., CTC of breast and lung cancer patients.

**Figure 2:**
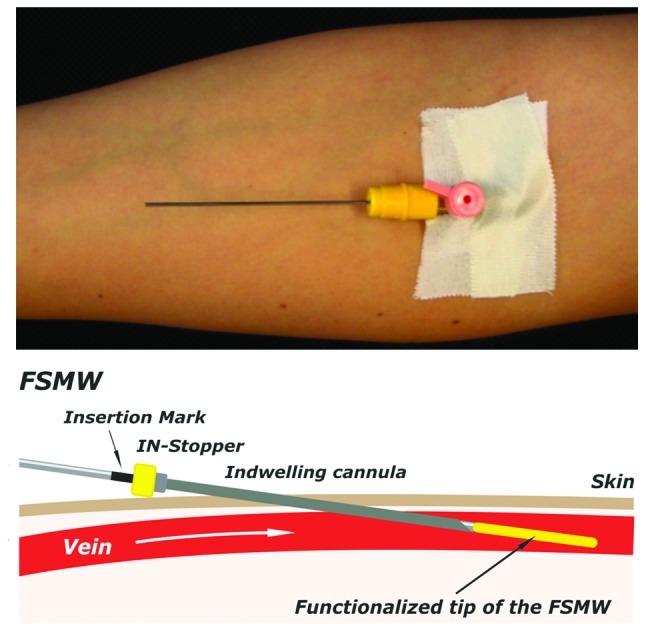
Insertion of the FSMW into the cubital vein through a conventional cannula. Above: arm bend showing the place where the FSMW is inserted into the cubital vein. Below: the FSMW is slowly pushed forward into the cannula until the anti-EpCAM-antibody-functionalized FSMW surface of 2 cm in length is exposed to the blood flow within the lumen of the vein. An IN-Stopper allows its secure fixation to the intravenous cannula. The correct length of insertion is indicated by a mark on the distal part of the FSMW, which is not inserted into the cannula. The FSMW remains in the cubital vein for 30 min.

The FSMW (Fig. 1) is based on a stainless steel medical wire (Seldinger guidewire) of 0.5 mm in diameter and 160 mm in length (EPflex, Dettingen, Germany). The first 20 mm are plated with a 2 μm thick gold layer deposited on the device by galvanization (OTEK, Brieselang, Germany). Subsequently, a hydrogel layer composed of a linear, synthetic polycarboxylate is attached to the gold layer (Xantec Bioanalytics, Düsseldorf, Germany). The carboxyl groups present in the hydrogel are then activated with EDC (1-ethyl-3-[3-dimethylaminopropyl] carbodiimide hydrochloride) and NHS (N-hydroxysuccinimide) (Sigma-Aldrich GmbH, Seelze, Germany) allowing for functionalization via covalent coupling of a chimeric antibody directed to the epithelial cell adhesion molecule CD326 (EpCAM; HEA 125, kindly provided by Dr G. Moldenhauer, German Cancer Research Center, DKFZ, Heidelberg, Germany) present on the surface of most CTC. Functionalization of the FSMW is carried out under clean room conditions. The FSMW is a sterile medical device and intended for single *in vivo* use only. For the *in vivo* application, the sterile FSMW is inserted into a standard 20G (pink color code) intravenous cannula (Fig. 2).

### In vitro experiments

**Figure 3:**
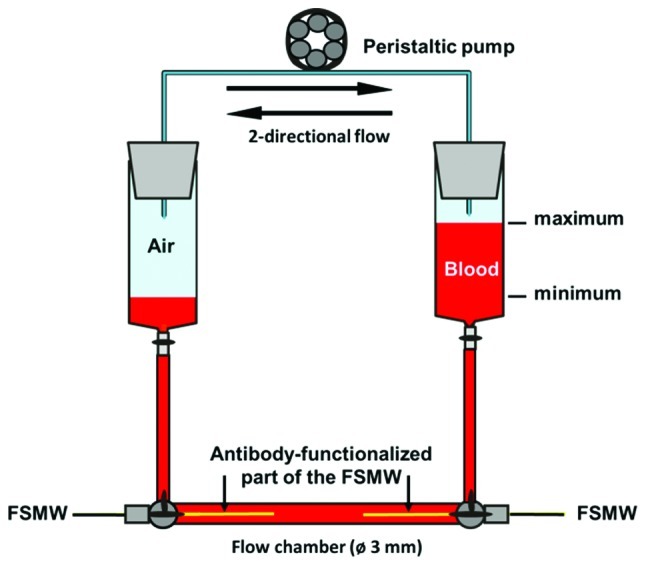
Schematic drawing of an *in vitro* flow system for repeated interaction of CTC with the FSMW. This *in vitro* flow system allows the simulation of *in vivo* venous blood flow conditions. A bidirectional flow is maintained by a peristaltic pump to enable repeated interaction of blood with the FSMW. This flow system is used to test interaction of CTC present in anti-coagulated blood or anti-coagulated blood spiked with cultured tumor cells with up to two FSMW simultaneously. The blood is not passing through the peristaltic pump to avoid mechanical impairment of cells. The flow system contains 16 ml of blood. Flow conditions are 20 ml/min, 44 cycles per 30 min, at RT.

Prior to FSMW application in patients, a hemodynamic flow system was applied (Fig. 3). Within this system, blood was routed via flexible tubes through a flow chamber into which up to two FSMW are inserted. The flow rate, velocity, and flow direction can be regulated by a peristaltic pump. A flow rate of 20 ml/min was applied to reflect *in vivo* flow conditions within cubital veins (21). In this *in vitro* flow system the FSMW was tested for binding of cultured EpCAM-positive SK-BR-3 breast cancer cells (CLS, Eppelheim, Germany), which were grown in cell culture flasks until cell monolayers reached a confluency of ∼80%. Adherent SK-BR-3 cells were detached with trypsin/EDTA (ethylene diamine tetraacetate) (Biochrom AG, Berlin, Germany), centrifuged, and suspended in phosphate-buffered saline (PBS). In addition, EDTA-anti-coagulated blood samples obtained from healthy donors were spiked with those cells and tested for binding to the FSMW. Furthermore, blood samples from breast or lung cancer patients were also assessed in the *in vitro* flow system to test for CTC binding to the FSMW.

### Cytotoxicity tests

To examine potential cytotoxic effects of the FSMW, we performed direct FSMW cell contact and material elution tests *in vitro*, based on the requirements for a class IIa medical device, as outlined in the ISO Guideline 10993-5 (www.iso.org). For the elution test, eluates of one or three FSMW devices (three to identify any variation in the production process), and of reference materials known to be toxic (copper wire, Goodfellow GmbH, Bad Nauheim, Germany) or non-toxic (Teflon wire, PTFE, Goodfellow GmbH) for normal human dermal fibroblasts (NHDF, C-12302, PromoCell GmbH, Heidelberg, Germany) were generated (0.095 m^2^ FSMW surface area eluted with 5 ml of RPMI-medium, FG 1235, Biochrom AG, at 37°C for 24 h), and their influence on cell morphology and viability of NHDF examined. Evaluation of potential effects of these eluates on cell morphology was done qualitatively by microscopic analysis for changes in cell morphology, cell adhesive capacity, and cell disintegration. Viability of cells was tested by use of the colorimetric TTC assay (triphenyltetrazolium chloride test).

In brief, for the microscopical inspection, NHDF were exposed to FSMW eluates for 48 h at 37°C. Eluates of reference materials known to be toxic (copper wire, Goodfellow GmbH) or non-toxic (Teflon wire, PTFE, Goodfellow GmbH) for NHDF were tested in parallel. After 48 h of incubation, cells were stained with the dye CFSE [5-(and-6)-carboxyfluorescein diacetate succinimidyl ester; Invitrogen, Carlsbad, USA] and inspected under the fluorescence microscope. Impairment of cell morphology was scored according to a common reaction index (none versus slight, moderate or severe).

To test for viability of NHDF, a colorimetric assay (TTC assay) was performed, which allows for the quantitative assessment of cell viability in the presence of material-derived eluates. After 48 h of incubation of NHDF with the eluates, a yellow tetrazolium compound (EZ4U, Cell Proliferation and Cytotoxicity Assay, Biomedica Medizinprodukte GmbH & Co. KG, Vienna, Austria) was added (3 h, 37°C) which was converted due to metabolic mitochondrial cellular activity to a brick-red formazan. Change in color was recorded at 450 nm by use of a microtiter plate reader (SPECTROstar-Omega, BMG Labtech, Ortenberg, Germany). For direct contact tests, the FSMW device was placed on a layer of adherent NHDF, followed by a 24 h incubation of this set-up at 37°C. Reference wires made of Teflon or copper were tested in parallel.

### Acute systemic toxicity

To assess potential hazardous effects of the FSMW in humans, possibly occurring, acute systemic toxicity of the FSMW was tested. In a parallel clinical study, aiming at the *in vivo* enrichment of trophoblasts from pregnant women, potential acute systemic toxicity of the eluates had already been monitored. According to these tests, no adverse effects were caused by the FSMW (22). For evaluation of the acute systemic toxicity of the FSMW, four test groups of five mice each [Charles River, Sulzfeld, Germany; NMRI (Han) mice, female non-pregnant, nulliparous, 19–24 g body weight, 4–5 weeks old) were injected intravenously or intraperitoneally with a single dose of four different extracts of the FSMW. The amount of eluates administered were adjusted to body weight at a volume of 50 ml/kg for 0.9% (w/v) NaCl, 5% (v/v) ethanol, and cotton seed oil, and at 10 g/kg for polyethylene glycol-400. Four control groups of five mice each were treated in the same manner with the corresponding extraction vehicle not previously exposed to the FSMW.

This dosing regime supplied an about 100 times higher dose of the FSMW than the expected dose in humans. The animals were followed up immediately after injection, and at 4, 24, 48 and 72 h intervals for body weight and toxic effects. Cage side observations included spontaneous activity, lethargy, recumbent position, convulsions, tremors, apnoea, asphyxia, vocalization, diarrhea, obvious changes in the skin and fur, eyes and mucous membranes (salivation, discharge). At the end of the observation period the animals were sacrificed. All animals were subjected to gross necropsy. Any gross pathological changes were recorded. The studies were approved by the Ethics Committee of the Medical University of Poznan, Poland.

### Hemocompatibility test

Objectives of the hemocompatibility test were assessment of the patient’s risk to develop thrombosis, coagulation, and hemolysis after *in vivo* exposure to the FSMW. The test method described in the ISO Guideline 10993-4:2002 (E) and according to Xu and coworkers (23) was employed. To assess any influence of the FSMW on the blood coagulation cascade, the plasma recalcification time was determined. The FSMW was incubated for 10 min at 37°C in citrate anti-coagulated platelet-poor plasma, which was then treated with CaCl_2_ to induce blood coagulation. Clotting time of plasma, which had not been exposed to the FSMW, served as a control. Fibrin formation was determined during a rigid up and down movement of the FSMW in a plasma sample or in a plasma sample which was not exposed to the FSMW. Time intervals until fibrin deposits were formed on the FSMW were recorded. For examination of any potential hemolytic risk exerted by the FSMW, erythrocytes were isolated from EDTA anti-coagulated blood of healthy donors and eluates of the FSMW added. After incubation at room temperature (RT), the erythrocyte-eluate-suspension was centrifuged and the hemolysis rate (% increase in cell-free hemoglobin concentration) determined photo-metrically. Products that have no hemolytic potential should exhibit a hemolytic rate of less than 5%.

To assess non-specific blood cell adhesion to the FSMW device, the antibody-functionalized part of the FSMW was put into an *in vitro* hemodynamic flow system (Fig. 3) which simulates the *in vivo* blood flow situation, including FSMW contact period, body temperature, and flow rate. After removal, the FSMW was inspected microscopically for blood cells deposited on the device. Then, in order to detect any fibrin deposits on the FSMW, the device was stained with antibodies directed to fibrin(ogen) [mouse anti-human fibrin antibody, IgM, 1:100 (2 μg/ml) in PBS; Santa Cruz Biotechnology Inc., Heidelberg, Germany] for 30 min, followed by the addition of secondary FITC-conjugated anti-IgM (Santa Cruz Biotechnology Inc.), 1:200 (2 μg/ml), for 30 min at RT. For detection of adherent platelets, a fluorescence-labeled antibody directed to CD41 was applied [anti-CD41a-PE, 1:100 (2 μg/ml) in PBS; Santa Cruz Biotechnology Inc.].

### Study population

Patients were recruited at the Wielkopolska Cancer Center, Department of Surgical Oncology and General Surgery, and at the Poznan University of Medical Sciences, both in Poznan, Poland. The healthy donor population was recruited at the Department of Obstetrics and Gynecology, Klinikum rechts der Isar, Technische Universitaet Muenchen, Munich, Germany. The studies were approved by the Institutional Ethics Committees and written informed consents of the patients and volunteers were obtained.

For the *in vitro* functionality test of the FSMW, we included 17 patients afflicted with breast cancer and 7 patients with NSCLC. For the *in vivo* functionality test of the FSMW, we included 12 breast cancer patients and 12 NSCLC patients (Tables I and II). Patients of both cancer types had different tumor stages and had not undergone surgery or received chemotherapy at the time of enrolment. Principal inclusion criteria for breast cancer and NSCLC patients were >18 years of age, histopathologically confirmed diagnosis of breast cancer or potentially resectable NSCLC with eligibility for radical surgery. Exclusion criteria: history of psychiatric disease, participation in other clinical trials, history of allergy, anaphylactic reactions, prior immunological diseases (anti-phospholipid antibody syndrome, Goodpasture’s syndrome, lupus erythematosus, polychondritis, rheumatoid arthritis, sarcoidosis, scleroderma, Sjogren’s syndrome, ANCA positive states), immunodeficiencies, prior infections with hepatitis viruses, the cytomegalovirus (CMV), or infectious diseases such as tuberculosis, syphilis, or toxoplasmosis. Principal exclusion criteria for the 29 healthy volunteers (premenopausal 24, postmenopausal 5) were >18 years of age, pregnancy or breastfeeding, any kind of oncological or allergic disease including asthma and thromboembolic complications. Median age of the volunteers was 27 (range 22–67).

For *in vitro* studies of the FSMW in an *in vitro* flow system, peripheral venous blood from patients afflicted with breast cancer or NSCLC was harvested into EDTA-tubes (Sarstedt AG & Co., Nuembrecht, Germany). Blood samples were kept at RT and processed in the flow system at RT within 72 h after drawing of the blood.

### In vivo application of the FSMW

The FSMW was designed to fit into a standard 20G intravenous cannula, which is placed into the cubital vein of a cancer patient or a healthy donor (Fig. 2). An IN-Stopper (Sarstedt AG & Co.) allows secure fixation to the intravenous cannula. The FSMW is slowly pushed forward into the cannula until the EpCAM-antibody-functionalized FSMW surface of 2 cm in length is exposed to the blood flow within the lumen of the vein. The correct insertion is indicated by a mark on the distal part of the FSMW, which is not inserted into the cannula. The FSMW remains in the cubital vein for 30 min. In the present study, the insertion of the FSMW was done before the respective patient underwent surgery of the primary tumor. During the procedure of FSMW application, the patient remained in a flat or supine position. The total volume of blood coming into contact with the FSMW during the 30 min application period is estimated at 1.5–3 liters (24).

### Inspection of the FSMW for bound CTC

After removal of the FSMW from the cubital vein, the FSMW was briefly and gently washed in PBS, followed by incubation in PBS containing 2% (w/v) bovine serum albumin (BSA, Carl Roth GmbH, Karlsruhe, Germany, purity grade ≥98%), for 30 min at RT. Characterization of CTC captured by the FSMW was done by immunocytochemical staining for EpCAM or cytokeratins 4, 5, 8, 9, and 18. Cells attached to the FSMW were incubated with an FITC-conjugated mouse monoclonal antibody directed to EpCAM [1:100 in PBS (10 μg/ml); Acris Antibodies GmbH, Herford, Germany] and a phycoerythrin (PE)-conjugated rabbit antibody raised against CD45 [1:25 in PBS (2 μg/ml); Life Technologies GmbH, Darmstadt, Germany]. Cells were counter-stained with the nuclear dye 4,6-diamidino-2-phenylindole (DAPI; 1 μg/ml PBS; Life Technologies GmbH). Intensity of the immunocytochemical staining of CTC was evaluated using an Axio Imager.A1m microscope (Zeiss, Jena, Germany) equipped with an AxioCam digital camera system and the AxioVision 4.6 software (Zeiss). EpCAM- or cytokeratin-positive cells included in the count had to disclose additional features such as a large cell body (diameter 10–50 μm), an irregular cell shape, a large irregularly shaped nucleus, and a high nuclear to cytoplasmic ratio (17,25,26). Cells were counted on each FSMW by an operator blinded to the clinical background of the patients. Results are given as number of CTC immobilized on the surface of the EpCAM-antibody-functionalized FSMW. In some cases, before inspection, cells were fixed with 4% (w/v) buffered paraformaldehyde.

## Results

Isolation and subsequent molecular characterization of CTC from the blood of cancer patients becomes increasingly important as it may serve as a ‘liquid biopsy’ with the potential of monitoring the course of the cancer disease and response to cancer therapy. For this purpose, but different than currently employed *ex vivo* CTC enrichment protocols, we applied a structured medical Seldinger guidewire (FSMW), functionalized with a chimeric monoclonal antibody directed to EpCAM, to be used *in vivo* to catch and enrich CTC from the peripheral blood pool (Fig. 1). The FSMW was first optimized *in vitro* for its CTC catching ability and then tested for biocompatibility according to the ISO guidelines for medical devices. Subsequently, suitability of the FSMW to catch and enrich CTC *in vivo* from circulating peripheral blood (Fig. 2) was tested in breast cancer and NSCLC patients in the framework of clinical trials, in comparison to healthy volunteers.

### In vitro evaluation of the CTC capture capability of the FSMW

**Figure 4:**
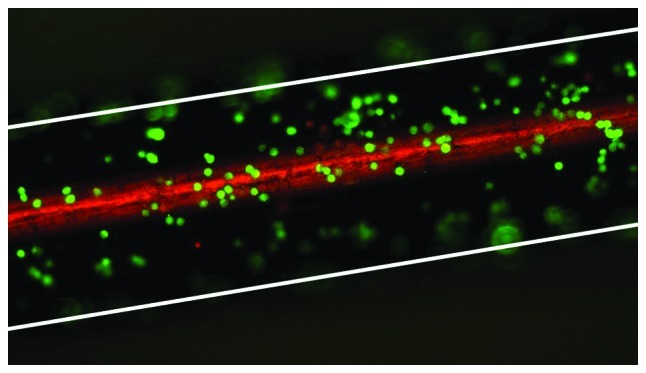
Representative image of cultured SK-BR-3 breast cancer cells captured by the FSMW. Cultured SK-BR-3 breast cancer cells were captured by the FSMW, fixed with buffered 4% (w/v) paraformaldehyde and then stained with FITC-labeled antibodies raised against EpCAM. The white lines denote the borders of the FSMW. Green fluorescence indicates SK-BR-3 cells. Note: view of the FSMW from the top focal plane, thus only cells attached here are in focus, cells in lower planes are not. The upper part of the FSMW appears in red due to fluorescence illumination of the gold layer.

Functionality of the FSMW in regard to its antibody-mediated CTC enrichment capability was tested in an *in vitro* dynamic flow system (Fig. 3) by capturing SK-BR-3 breast cancer cells simulating human CTC which had been added to 20 ml of anti-coagulated blood of healthy volunteers or blood obtained from breast cancer or NSCLC patients. The intention of these experiments was to test: a) whether EpCAM-antibody mediated tumor cell immobilization on the FSMW occurs under conditions similar to venous blood flow and b) whether non-malignant blood cells are interfering with the capture capability of the FSMW. Representative microscopic images of FSMW-immobilized fluorescence-labeled SK-BR-3 cells from the *in vitro* flow system experiments are shown in Fig. 4. The functionality of the FSMW in the *in vitro* flow system was also tested with blood samples from breast cancer and NSCLC patients. Under these conditions, the FSMW captured CTC in 7 out of 17 (41%) anti-coagulated blood samples of breast cancer patients (range 1–44, median 5). Also, all anti-coagulated blood samples of NSCLC patients (n=7) turned out to be positive for CTC (range 1–8, median 7).

### Biocompatibility of the FSMW device

**Figure 5:**
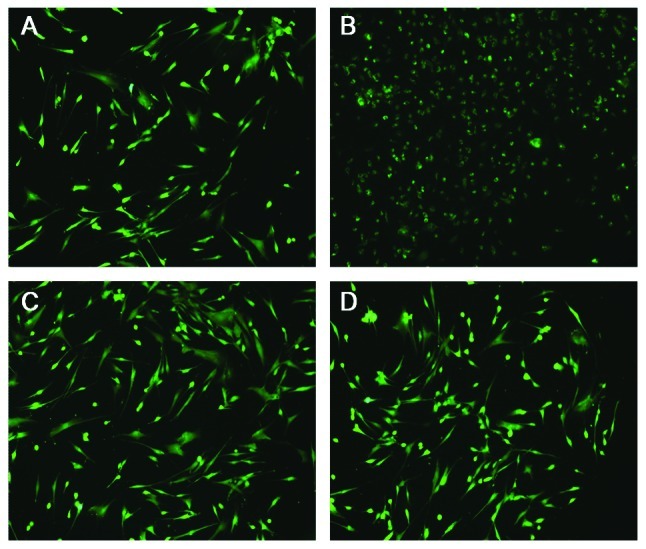
Test for potential adverse effects of eluates obtained from different wire materials on cell integrity by assessment of cell morphology; according to ISO guideline 10993-5. Cultured CFSE-stained NHDF were incubated with different wire eluates for 24 h at 37°C to demonstrate any influence of the eluates on cell morphology. (A), Reference Teflon wire eluate with known non-cytotoxic effect on NHDF: no apparent changes in cell morphology. (B), Reference copper wire eluate with known cytotoxic effect on NHDF: severe changes in cell morphology. (C), FSMW eluate prepared from a single FSMW: no apparent change in cell morphology. (D), FSMW eluate prepared from three FSMW: no apparent change in cell morphology. Staining of NHDF with CFSE was performed to improve microscopic inspection.

**Figure 6:**
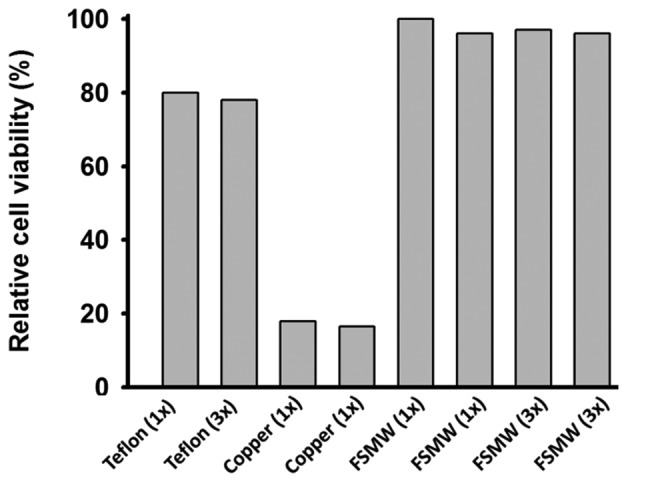
Test for potential adverse effects of eluates obtained from different wire materials by TTC assay according to ISO Guideline 10993-5. The ability of vital cells to convert a tetrazolium salt into a red formazan derivative was employed to determine the influence of different wire eluates on the viability of cultured NHDF. Reference eluates prepared from known non-cytotoxic (Teflon) and cytotoxic (copper) wire materials and from eluates prepared from different FSMW were added to cultured NHDF and formazan production recorded photometrically at 450 nm after 3 h of incubation at RT. Obtained optical density values are expressed as relative cell viability in percent. Eluates were prepared from indicated numbers of test materials. Formazan production by NHDF in the absence of any test wire eluates was set to 100%.

To demonstrate the biocompatibility and safety of the FSMW for cancer patients or healthy volunteers during its *in vivo* application, tests were performed according to ISO guidelines recommended for class IIa medical devices. Eluates prepared from the FSMW as described in Materials and methods were tested for their potential effects on the viability of cultured NHDF. Microscopic inspection of NHDF after treatment with FSMW or Teflon wire eluates did not disclose any changes in cell morphology. In contrast, NHDF subjected to copper wire extract, detached from the cell culture dish substratum and assumed a spindle-shaped cell morphology (Fig. 5). Cellular mitochondrial activity (as a surrogate marker for cell viability) assessed by the TTC test remained unchanged when NHDF were exposed to the FSMW eluate, compared to that observed in NHDF treated with copper wire eluate, resulting in a more than 80% reduction of cell viability (Fig. 6). Direct contact of NHDF with the FSMW gave similar results. Even after a 24-h incubation period of the FSMW on cultured NHDF, no perturbation of cell membrane integrity was observed. In contrast, the copper wire as a positive control had a negative influence on NHDF since they detached from the substrate and showed a reduced proliferation rate.

Tests for *in vivo* biocompatibility of the FSMW did not reveal any signs of acute toxicity when FSMW eluates (n=4) were injected intravenously or intraperitoneally into NMRI mice (n=5; for details see Materials and methods). No FSMW-related mortalities were recorded. All mice survived the test period of 72 h independent of whether a negative control or a FSMW eluate was applied and the mice showed normal food intake and unchanged body weight.

Hemocompatibility tests did not indicate any hemolytic effects of FSMW eluates. The re-calcification time of platelet-poor plasma of citrate anti-coagulated blood from healthy donors in the presence of the FSMW (n=5) was comparable to the recalcification time of citrate anti-coagulated blood which had not been exposed to the FSMW (n=5).

### In vivo application of the FSMW in healthy volunteers and in breast cancer and NSCLC patients

**Figure 7:**
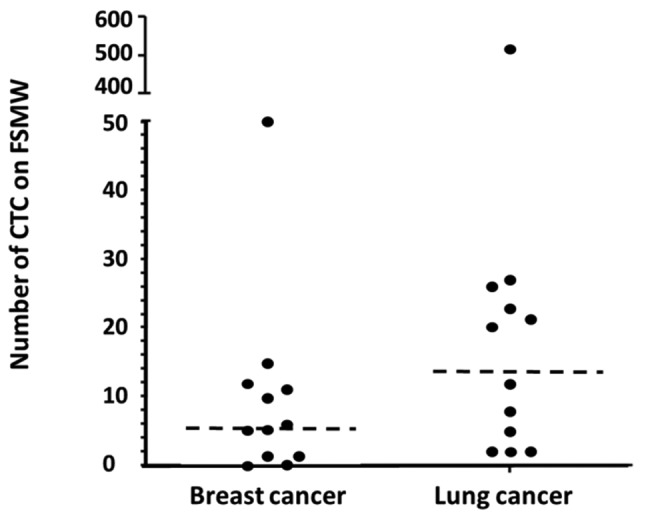
Number of CTC captured by the FSMW *in vivo*. Twelve NSCLC and 12 breast cancer patients were exposed to the FSMW *in vivo* for 30 min and the number of CTC attached to the FSMW enumerated. Inclusion criteria for identification of CTC: EpCAM-positive, pan-cytokeratin-positive, CD45-negative, positive staining of nuclei with DAPI. Median values of cell counts (horizontal bar): breast cancer 5.5, lung cancer 16. See also Table III.

**Figure 8:**
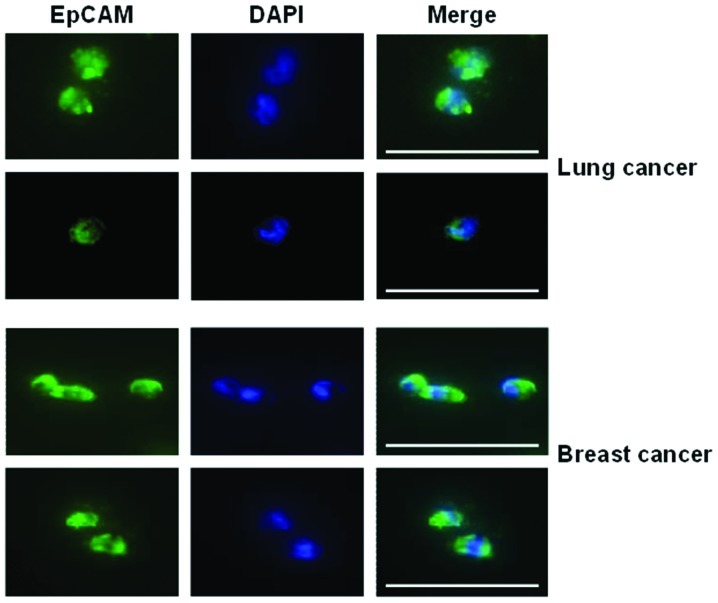
Representative images of CTC isolated by the FSMW *in vivo* from the peripheral blood of lung and breast cancer patients. NSCLC patients (upper panel); breast cancer patients (lower panel). CTC enriched by the FSMW were identified via secondary staining for EpCAM using an FITC-conjugated mouse monoclonal antibody directed to EpCAM (green) and nuclear stain (DAPI, blue). The absence of white blood cells (no staining) was confirmed by exposure of the cells to a PE-labeled rabbit antibody directed to CD45. Scale bar, 50 μm.

The FSMW was inserted into the cubital veins of 29 healthy volunteers, 12 patients afflicted with breast cancer and 12 patients presenting with NSCLC. This invasive procedure was approved by the local Ethics Committees (Poznan, Munich). All healthy volunteers and patients tolerated the short-term (30 min) *in vivo* exposure to the FSMW without any signs of adverse events. No CTC or other epithelium-derived cells were detectable on the FSMW applied to healthy volunteers. The FSMW captured CTC in 10 out of 12 patients afflicted with breast cancer (83.3%) with a median of 5.5 CTC (range 1–50) and a mean of 9.7±14 per FSMW. In all of the 12 NSCLC patients assessed, CTC were detectable on the FSMW with a median of 16 CTC (range 2–515) and a mean of 55±145 (Table III). Thus, for the 24 cancer patients tested, the CTC detection rate was 91.6% (Fig. 7). Representative images of FSMW-captured CTC from breast cancer patients stained for EpCAM expression are depicted in Fig. 8.

In a series of clinical trials employing the CellSearch device (Veridex LLC, Warren, NJ) as an immunomagnetic isolation technique for CTC in metastatic breast cancer patients (14,27), a disease-specific cut-off was used to define patient groups with a favorable (<5 CTC per 7.5 ml of blood) and an unfavorable prognosis (≥5 CTC/7.5 ml of blood). Basic characteristics of the NSCLC and breast cancer patients enclosed in the present observatory study and the respective number of CTC captured *in vivo* by the FSMW are shown in Tables I and II. Squamous cell lung carcinoma (8 out of 12 cases) was the most frequent NSCLC subtype investigated. In the peripheral blood of these patients the FSMW captured ≥5 CTC in 5 of the 8 cases (62.5%). Furthermore, in 66.7% of the 12 NSCLC patients with a histological grading G2 >5 CTC were captured by the FSMW. Invasive-ductal carcinoma was the most common subtype of breast cancer in 58.3% (7 out of 12) of the cases; 85.7% of these cases (n=6) displayed more than 5 CTC. In breast cancer patients with positive estrogen and/or progesterone receptor status of their primary tumors (n=10), the FSMW captured ≥5 CTC in 70% (n=7) of the cases. We would like to mention that all of the lung and breast cancer patients covered a variety of stages (Tables I and II), but only one of the 24 patients presented with distant metastasis (breast cancer, T1N1M1) at the time of FSMW application.

## Discussion

Metastases rather than the primary tumor are the main cause of death from cancer (28). Although there is still no comprehensive knowledge of the biology of the metastases that actually would need treatment, several studies indicate important molecular differences between primary tumor and metastases at the gene and protein level (29). The differential expression of biomarkers between primary tumor and metastases with proven clinical relevance could imply that molecular features of metastatic tumor cells do have a superior predictive value over looking at the primary tumor cells alone (30). Still, identification of cancer biomarkers for clinical response in tumor cells and a better understanding of the mechanisms involved in drug sensitivity would require repeated biopsies from metastatic lesions.

Yet, only a minority of metastatic lesions is resected, so most histopathologic studies investigated primary tumor tissues only (29). This attitude is based on the fact that taking biopsies is associated with an increased risk of complications and often painful discomfort for the cancer patient, and it is only feasible in patients with easy-to-access lesions. A markedly interesting alternative to taking biopsies from metastatic lesions is collecting CTC from the peripheral blood pool of cancer patients. After cancer cells escape from primary tumor tissues, they intravasate by lympho-hematogenous dissemination to distant sites of the body, including the bone marrow and the blood (17,31). In contrast to CTC disseminated to the bone marrow, CTC can be easily obtained and enriched from the peripheral blood (28,32). For this, robust and reproducible laboratory techniques are needed for blood-borne CTC enrichment and enumeration. In recent years, antibody-based *ex vivo* cytometric methods of tumor cell enrichment and detection have become the standard to identify CTC. Nonetheless, in the past, also nucleic acid-based detection approaches were frequently used, with the disadvantage that CTC enumeration or assessment of cell morphology is not possible by this technology (28,30,33–37).

Different from these methods, we have developed a novel, proprietary *in vivo* CTC detecting technology which makes use of a structured, FDA-cleared (510k) Seldinger guidewire. Seldinger guidewires are otherwise used for angiography, insertion of chest drains, and central venous catheters. This novel functionalized and structured Seldinger guidewire (FSMW) is coated at its tip (2 cm of length) with a hydrogel layer to which chimeric antibodies directed to epithelial cell adhesion molecule (EpCAM) are covalently attached. The FSMW is placed into the cubital vein of a cancer patient to allow *in vivo* binding of rare CTC from the patient’s entire circulating blood pool of several liters. This approach is different from any of the other *ex vivo* CTC enriching technologies which are detecting CTC in a limited quantity of blood, usually a few milliliters only (28,33,38). In this respect, one should keep in mind that CTC in the peripheral blood of cancer patients exist in extreme rarity and can be as low as one CTC per 10^5^–10^7^ of other blood cells in advanced disease stages, with even lower numbers in the blood of early-stage-disease patients (35,39,40). While it is understandable that CTC are present in advanced, metastasized cancer, it is not clear how early in the tumor cell invasion and dissemination process they will occur in the blood circulation.

Our novel FSMW may help to clarify if CTC not only are predictors of patient outcome in the metastatic phase of cancer of e.g., the breast, ovary, kidney, lung, colon, and head and neck (27,33,41–47), but also in earlier stages of the cancer disease (48–52). This is done in the context of two ongoing cancer therapy trials, in advanced lung (ISRCTN55277999) and early and advanced stage breast cancer patients (ISRCTN66203697). Within these clinical therapy trials, enumeration of CTC is not a primary endpoint; the FSMW was employed solely to test the feasibility and performance of the medical device *in vivo*, but in context of prospective clinical cancer trials. The FSMW proved to be a non-hazardous, safe medical *in vivo* device with no adverse effects on cell viability in the mandatory *in vitro* tests. Moreover, no harmful impact of the FSMW on the blood coagulation system after the short *in vivo* exposure time of 30 min was observed, neither in breast/lung cancer patients nor in healthy volunteers.

Different from automated *ex vivo* CTC enrichment systems, the novel FSMW allows direct microscopic control of tumor cells bound to the antibody-labeled hydrogel of the device. Similar to other antibody-based technologies, CTC bound to the FSMW are subjected to subsequent *ex vivo* labeling with antibodies directed to CD45 to identify any non-specifically attached leukocytes, and with DAPI, to visualize the nucleus of the cells. Once attached to the FSMW, CTC are fixed to allow identification with fluorescence-labeled antibodies directed to cytokeratins and further fluorescent staining for other cellular biomarkers, such as HER2, estrogen/progesterone receptor, epidermal growth factor (EGF)-receptor, urokinase-type plasminogen activator (uPA)/plasminogen activator inhibitor type-1 (PAI-1), and other cancer biomarkers of interest.

Other *ex vivo* CTC enrichment methods rely on the assumption that tumor cells are different in cell density and dielectric properties or of relative larger size than the majority of blood cells (28,33,38,53,54), yet, not allowing an immediate visual microscopic control of the enriched CTC. Some technologies are using microfluidic filters or magnetic beads, also coated with antibodies directed to EpCAM, in some cases combined with magnetic rods, microposts, or herringbones to catch the antibody-labeled CTC (28,38). Yet, all these EpCAM-antibody-based technologies assume that tumor cells of epithelial origin do express the EpCAM antigen. This is not always so, epithelial cells may lack the EpCAM antigen. In this case, additional antibodies to other epithelial surface antigens should be considered for cell trapping, such as CD49f, HER2, MUC1/2, or carcinoembryonic antigen (CEA) (34,55). The novel FSMW is also making use of trapping epithelial-derived tumor cells with a high-affinity antibody directed to EpCAM but other cell surface-directed antibodies can be easily covalently attached to the hydrogel of the FSMW, alone or in combination with EpCAM-directed antibodies. That way, not only tumor cells of epithelial origin, but also CTC derived from malignant melanomas, sarcomas, and other types of cancer can be trapped. Even further, antibodies can be attached to the FSMW which are targeting circulating non-tumor cells such as endothelial cells, rare forms of leukemia cells, or trophoblast cells of pregnant women. Indeed, in another clinical cell trapping approach employing a FSMW covalently modified with antibodies directed to HLA-G (a trophoblast surface antigen), circulating trophoblasts were caught from the peripheral blood of pregnant women and subjected to testing of genetic fetal abnormalities.

Recent results by Farace *et al* point to important discrepancies between the numbers of CTC enumerated by different enrichment technologies, also depending on the type of tumor (56). Specifically, Flores *et al* showed for breast and lung cancer patients that even for one type of CTC enrichment technology, simply by using two different cell enrichment kits (CellSearch Epithelial Kit versus CellSearch Profile Kit) on the Veridex CellSearch™ machine, up to 20-fold differences in CTC yield were obtained by using the CellSearch Profile Kit (57). Thus, keeping these results in mind, further preclinical studies are needed to compare performance and yield of the novel *in vivo* FSMW CTC enrichment technology with other, established *ex vivo* CTC enrichment technologies.

## Figures and Tables

**Table I:** Clinical characteristics of breast cancer patients assessed for CTC enrichment *in vivo* and histomorphological features of the primary tumor.

Classification	No. of patients	Patients with 1–4 CTC (%)	Patients with ≥5 CTC (%)
All patients	12	3 (25)	9 (75)
ER-/PR-status			
Positive for either	10	3 (30)	7 (70)
Negative for both	2	0	2 (100)
HER2-status			
Positive	3	0	3 (100)
Negative	9	3 (33.3)	6 (66.7)
Histological classification			
Invasive-ductal	7	1 (14.3)	6 (85.7)
Invasive-lobular	3	1 (33.3)	2 (66.7)
Invasive-ductal/invasive lobular	1	1 (100)	0
Invasive-ductal/bifocal cancer	1	0	1 (100)
Adjuvant therapy			
Chemotherapy	3	1 (33.3)	2 (66.7)
Endocrine therapy	1	0	1 (100)
No adjuvant therapy	8	2 (25)	6 (75)
Tumor stage			
T1N1M0	2	1 (50)	1 (50)
T1N3M0	1	0	1 (100)
T2N1M0	3	1 (33.3)	2 (66.7)
T2N3M0	1	0	1 (100)
T4N0M0	1	0	1 (100)
T4N2M0	1	0	1 (100)
T4N+N/A	1	0	1 (100)
T1N1M1	1	1 (100)	0
T3N+N/A	1	0	1 (100)

**Table II:** Clinical characteristics of NSCLC patients assessed for CTC enrichment *in vivo* and histomorphological features of the primary tumor.

Classification	No. of patients	Patients with 1–4 CTC (%)	Patients with ≥5 CTC (%)
All patients	12	2 (16.7)	8 (66.7)
Histological classification			
Squamous cell lung carcinoma	8	1 (12.5)	5 (62.5)
Adenocarcinoma	3	1 (33.3)	2 (66.7)
Large cell lung carcinoma	1	0	1 (100)
Histological grade			
G2	9	2 (22.2)	6 (66.7)
G3	2	0	1 (50)
Unknown	1	0	1 (100)
Tumor stage			
T2N0M0	4	1 (25)	2 (50)
T2N1M0	3	0	3 (100)
T2N2M0	2	1 (50)	1 (50)
T3N0M0	1	0	1 (100)
T3N1M0	2	0	1 (50)

**Table III:** CTC enriched by *in vivo* use of the FSMW: summary for lung and breast cancer patients.

	Breast cancer (%)	NSCLC (%)
No. of patients	12	12
Positive	10 (83.3)	12 (100)
CTC on FSMW		
Range	0–50	2–515
Median	5.5	16
Mean ± SD	9.7±13.7	55.4±145
CTC counts		
0	2 (16.7)	0
<5	2 (16.7)	3 (25)
≥5	8 (66.7)	9 (75)

SD, standard deviation.
